# Neuroprotective potential of *Erigeron bonariensis* ethanolic extract against ovariectomized/D-galactose-induced memory impairments in female rats in relation to its metabolite fingerprint as revealed using UPLC/MS

**DOI:** 10.1007/s10787-023-01418-3

**Published:** 2024-01-31

**Authors:** Weam W. Ibrahim, Rabab H. Sayed, Mohamed F. Abdelhameed, Enayat A. Omara, Mahmoud I. Nassar, Noha F. Abdelkader, Mohamed A. Farag, Abdelsamed I. Elshamy, Sherif M. Afifi

**Affiliations:** 1 Department of Pharmacology and Toxicology, Faculty of Pharmacy, Cairo University, Kasr El-Aini St., Cairo, 11562 Egypt; 2 Pharmacology Department, National Research Centre, Dokki, 12622 Giza Egypt; 3 Pathology Department, National Research Center, Dokki, Cairo, 12622 Egypt; 4 Natural Compounds Chemistry Department, National Research Centre, Dokki, 12622 Giza Egypt; 5 Pharmacognosy Department, Faculty of Pharmacy, Cairo University, Kasr El Aini St., Cairo, 11562 Egypt; 6 Pharmacognosy Department, Faculty of Pharmacy, University of Sadat City, Sadat City, 32897 Egypt

**Keywords:** *Erigeron bonariensis*, Chemical profiling, Alzheimer’s disease, α7-nAChR, Jak2/STAT3/PI3K/GSK-3β

## Abstract

*Erigeron bonariensis* is widely distributed throughout the world's tropics and subtropics. In folk medicine, *E. bonariensis* has historically been used to treat head and brain diseases. Alzheimer’s disease (AD) is the most widespread form of dementia initiated via disturbances in brain function. Herein, the neuroprotective effect of the chemically characterized *E. bonariensis* ethanolic extract is reported for the first time in an AD animal model. Chemical profiling was conducted using UPLC–ESI-MS analysis. Female rats underwent ovariectomy (OVX) followed by 42 days of D-galactose (D-Gal) administration (150 mg/kg/day, i.p) to induce AD. The OVX/D-Gal-subjected rats received either donepezil (5 mg/kg/day) or *E. bonariensis* at 50, 100, and 200 mg/kg/day, given 1 h prior to D-Gal. UPLC–ESI-MS analysis identified 42 chemicals, including flavonoids, phenolic acids, terpenes, and nitrogenous constituents. Several metabolites, such as isoschaftoside, casticin, velutin, pantothenic acid, xanthurenic acid, C18-sphingosine, linoleamide, and erucamide, were reported herein for the first time in Erigeron genus. Treatment with *E. bonariensis* extract mitigated the cognitive decline in the Morris Water Maze test and the histopathological alterations in cortical and hippocampal tissues of OVX/D-Gal-subjected rats. Moreover, *E. bonariensis* extract mitigated OVX/D-Gal-induced Aβ aggregation, Tau hyperphosphorylation, AChE activity, neuroinflammation (NF-κBp65, TNF-α, IL-1β), and apoptosis (Cytc, BAX). Additionally, *E. bonariensis* extract ameliorated AD by increasing α7-nAChRs expression, down-regulating GSK-3β and FOXO3a expression, and modulating Jak2/STAT3/NF-ĸB p65 and PI3K/AKT signaling cascades. These findings demonstrate the neuroprotective and memory-enhancing effects of *E. bonariensis* extract in the OVX/D-Gal rat model, highlighting its potential as a promising candidate for AD management.

**Graphical Abstract:**

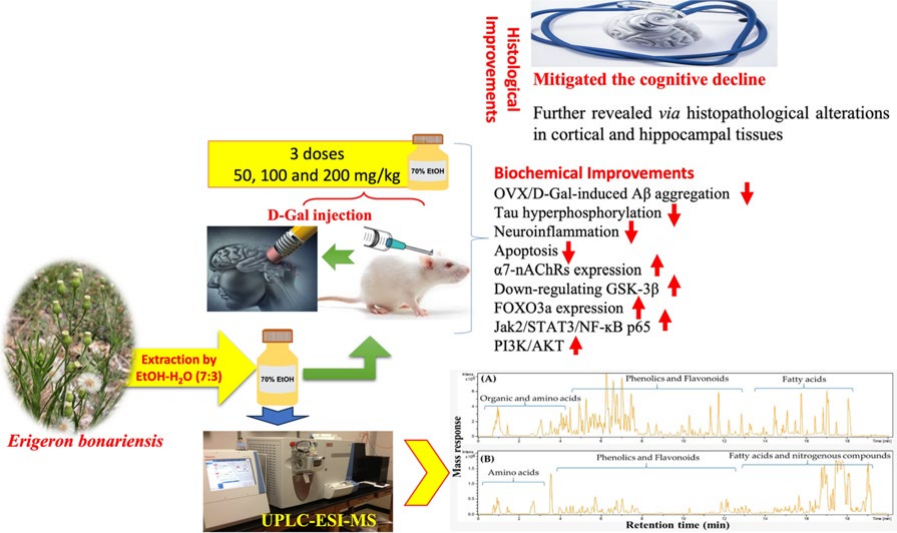

## Introduction

Alzheimer's disease (AD) is a progressive neurodegenerative disorder accounting for most cases of age-related dementia (Feigin et al. 2019). Although the etiology of AD is still unknown, many factors have been identified as major contributors to the disease, including amyloid-β (Aβ) deposition, tauopathy, oxidative stress, neuroinflammation, and increased activity of apoptotic pathways (Kinney et al. 2018; Villemagne et al. 2018). Long-term administration of D-galactose (D-Gal) to ovariectomized (OVX) rats serves as an animal model that imitates behavioral, biochemical, and pathological alterations in AD (Hua et al. 2007; Kamel et al. 2018; Ibrahim et al. 2019).

Alpha 7 nicotinic acetylcholine receptors (α7-nAChRs) are ligand-gated ion channels that are highly expressed in brain regions involved in the regulation of cognitive function (Ma and Qian 2019). The α7-nAChR has been shown to play a vital role in the pathogenesis of the early phase of AD (Takata et al. 2022). Of note, Aβ accumulation has been reported to directly inhibit α7-nAChRs and underpin cognitive decline in AD patients (Nakaizumi et al. 2018; Potasiewicz et al. 2020). Thus, activation of α7-nAChRs exerts cognitive-enhancing effects via various mechanisms, including stimulating the cholinergic pathway, regulating inflammation and apoptosis, and attenuating the effects of Aβ (Hoskin et al. 2019). The α7-nAChRs-mediated neuroprotection against Aβ is initiated via activating Jak2 and modulating its downstream signaling cascades, including STAT3, PI3K, and GSK-3 (Marrero and Bencherif 2009; Ma and Qian 2019).

Asteraceae plants are widely distributed worldwide with extensive reports on their use in the treatment of several diseases (Elgamal et al. 2021). *Conyza* species (Family Asteraceae), compromising approximately 150 plants (Wang et al. 2018), have significant medicinal uses such as treating rheumatism, diarrhea, toothache, hemorrhoids, bleeding, and skin injuries (Ayaz et al. 2017; Peng et al. 2020; Elgamal et al. 2021). *Erigeron bonariensis* L. (previous name: *Conyza linifolia* (Willd.) Täckh.) is a unique plant in tropical and subtropical areas (Gabr 2021) that has not been previously reported regarding its chemical profiling. The chemical composition of its essential oil revealed its richness in terpenoids, including monoterpenes and sesquiterpenes (Harraz et al. 2015; Elgamal et al. 2021), exhibiting antibacterial, insecticidal (Harraz et al. 2015), anticancer, and anti-aging effects (Elgamal et al. 2021). Recently, Peralta et al. (2022) reported on the isolation of highly oxygenated compounds, including fatty acids, monoterpenes, phenolic acids, and flavonoids from *E. bonariensis* extracts, with significant phytotoxic effects.

Therefore, the present study aimed to: (i) determine the chemical composition of *E. bonariensis* aerial parts ethanolic extract via ultra-performance liquid chromatography–electrospray ionization-tandem mass spectrometry (UPLC–ESI-MS) in an untargeted manner to characterize a broad range of polar and non-polar metabolites in a holistic manner (Farag et al. 2022); (ii) assess the protective role of *E. bonariensis* extract against OVX/D-Gal-induced memory impairments in rats; and (iii) unveil the mechanistic pathways of *E. bonariensis* neuroprotective effects, focusing on the role of α7-nAChR as one of the main nAChR subtypes that is extensively expressed in brain area implicated in learning and memory processes.

## Materials and methods

### Plant collection

Fresh aerial parts of *E. bonariensis* were collected from Cairo–Alexandria Desert Road, Egypt during the stage of plant flowering on the 10th of April 2021, early morning at 6:00 am. The plant collection and authentication were kindly performed by Prof. Ahmed M. Abdel Gawad, Professor of Plant Ecology, Mansoura University, Egypt. The plant identification was performed as previously described (Boulos 2002). A plant specimen [EB(x215)-YD-20197-021] was saved in the herbarium of Mansoura University, Egypt. The collected plant parts were left for complete drying in a shaded, clean open-air place at ± 25 °C for 15 days, and then crushed into powder.

### Extraction process

The plant powder (1200 g) was extracted on cold using a mixture of ethanol and bi-distilled water at a ratio of 7:3 for 3 consecutive days at ± 25 °C followed by filtration. This process was repeated twice, and the whole extract was dried under reduced pressure at 45 °C using a rotary evaporator (Heidolph Laborota 4003, Germany) till complete drying. The extract was obtained as a black gum (46.5 g) and was kept in the fridge (4 °C) inside a dark black glass vial until further analysis.

### Ultra-performance liquid chromatography–electrospray ionization-high-resolution tandem mass spectrometry profiling of extract

After extraction, the UPLC–ESI-MS profiling of the plant extract was performed using an Acquity UPLC system (Waters, Germany) equipped with an HSS T3 column (100 × 1.0 mm, particle size 1.8 µm; Waters), applying the same parameters reported by Ayoub et al. (2022), Hassan et al. (2023), and Abib et al. (2023).

### Drugs and chemicals

D-Gal and donepezil were purchased from Sigma-Aldrich Chemical Co., St. Louis, MO, USA, and dissolved in saline. A high analytical grade of other chemicals was used.

### Animals

Three-month-old female Wistar rats, weighing 160–190 g, were acquired from the animal house of the National Research Centre (Giza, Egypt). They were habituated for 1 week before starting the experiment at the animal facility of Faculty of Pharmacy, Cairo University (Cairo, Egypt). Rats were supplied with food and water ad libitum and housed in monitored environmental conditions of temperature (23 ± 2 °C), humidity (60 ± 10%), and 12/12 h light/dark cycle. The investigational procedures were reviewed and accepted by the Ethics Committee of Faculty of Pharmacy, Cairo University (Ethical approval no: PT3352). The protocol also followed the guidelines of the National Institutes of Health Guide for Care and Use of Laboratory Animals (2011). Every attempt was made to reduce the suffering of animals through the experiments.

### Experimental design

Sixty female rats were arbitrarily distributed between six groups (*n* = 13/group). Group I (SO): sham operation (SO) was conducted on rats and served as the control group. In group II (OVX/D-Gal), bilateral ovariectomy (OVX) was performed on rats according to the operation method discussed by Salama et al. (2021) and after a recovery period of 5 days, they received daily intraperitoneal injection of D-Gal (150 mg/kg) for 42 days (Ibrahim et al. 2022; El Sayed et al. 2023). Group III (Donepezil): OVX/D-Gal-subjected rats received donepezil (5 mg/kg/day) (Ademosun et al. 2022) orally for 42 days, given 1 h prior to D-Gal administration. Group IV, V, and VI (*E. bonariensis* 50, 100, and 200 mg/kg/day): OVX/D-Gal-subjected rats received the alcoholic extract of *E. bonariensis* at three different doses (50, 100, and 200 mg/kg/day) (Barua et al. 2019; El-Akhal et al. 2021) orally for 42 days, given 1 h prior to D-Gal administration. Before the end of the experiment by 4 days, all animals were subjected to the Morris water maze (MWM) test for memory performance evaluation. The MWM was performed over 4 successive days. The training phase was conducted on days 39–41 (first 3 days), and 24 h after the last training session, the probe trial was performed on day 42 (4th day). One day later after performing the probe test (day 43), rats were decapitated under anesthesia. Brains were rapidly excised, washed, and dried and then weighed. The hippocampi were separated from their brains and flash frozen in liquid nitrogen, and then stored at – 80 °C for later biochemical analysis. Based on the behavioral and histopathological examination of the three tested doses of *E. bonariensis* alcoholic extract, the dose 100 mg/kg was selected and used for further biochemical assessment.

### Ovariectomy

Rats were exposed to OVX under anesthesia using ketamine (50 mg/kg, i.p.) and xylazine (10 mg/kg, i.p.). In brief, the area located on each lateral side of the abdomen between the last rib and the hip was shaved and disinfected, and then, a small opening was made in this area exposing the ovaries and its associated oviducts. Afterward, a hemostatic clamp was positioned underneath the ovaries and a suture knot was done below it, and then, the ovaries were cut with sterile scissors. Using absorbable and non-absorbable threads, the muscle and skin layers were sutured. The SO was done as previously illustrated except for ovarian removal. An antibiotic spray and anti-inflammatory cream were applied to the wound. Rats were given chow devoid of soy to ignore the impact of phytosteroids (Khajuria et al. 2012; Ibrahim et al. 2016; Salama et al. 2021).

### Behavioral assay

The MWM assesses the spatial reference memory in rodents. The maze is a four-equally divided circular pool with a diameter of 150 cm and a height of 60 cm and filled with 40 cm deep opaque water containing a non-toxic water-soluble black dye. A platform of 8 cm diameter was located just under the water surface in the center of a certain quadrant. In each day of the three training days, 2 training trials (120 s each) were given for each rat. In the training session, each animal was freely left to locate the platform. When the animal succeeded to reach the platform, it could stay on it for 10 s. However, when the animal failed to get to the platform during the specified time, it was gently guided to the platform and left on it for 30 s. 1 day following the last training, the platform was removed to conduct the probe test where each rat was left in the pool for 60 s. During this period, animals’ performance was videotaped by an overhead camera and then analyzed using the ANY-Maze video tracking software (Stoelting Co, USA) (Ibrahim et al. 2020).

### Enzyme-linked immunosorbent assay

In each of the SO, OVX/D-Gal, Donepezil, and *E. bonariensis* 100 mg/kg groups, the hippocampi of 6 rats were homogenized in ice-cold phosphate-buffered saline. Rat-specific ELISA kits acquired from My BioSource (San Diego, CA, USA) were utilized to assess the hippocampal content of Aβ42 (Cat. #MBS726579), Cytc (Cat. #MBS727663), NF-κBp65 (Cat. #MBS775083), and IL-1β (Cat. #MBS825017). Additionally, BCL-2 and BAX hippocampal contents were measured using rat ELISA kits provided by Biomatik (Ontario, Canada, Cat. # EKC40527 and EKC41377, respectively). Further, AChE was determined using an ELISA kit supplied by CUSABIO Technology LLC, China (Cat. # CSB-E11304r) and TNF-α quantification was performed using the PicoKine ELISA kit (Boster, CA, USA, Cat. #MBS175904). All procedures were performed according to the manufacturer’s guidelines. The protein content of tissue homogenates was determined as previously described (Bradford 1976).

### Western blot

The hippocampal protein expression of phosphorylated forms of Jak2 and STAT3 was determined in each group of SO, OVX/D-Gal, Donepezil, and *E. bonariensis* 100 mg/kg groups using Western blot technique. In brief, the protein content of the right side of hippocampal tissues (*n* = 3/group) was extracted by Ready Prep^™^ protein extraction kit, Bio-Rad Inc., CA, USA, and then assessed as previously described (Bradford 1976). Equal protein amounts were loaded onto SDS-polyacrylamide gel for their electrophoresis separation according to the molecular weight, and then, they were transported into a nitrocellulose membrane. After being soaked in 5% skimmed milk, the blocked membranes were incubated at 4°C overnight on a roller shaker with solutions containing the following primary antibodies provided by Cell Signaling Technology, USA: anti-p-Jak2 (Tyr1007/1008), anti-p-STAT3 (Tyr705), and anti-β-actin antibodies (Cat. #3771, 9131, and 4967, respectively). After washing the membranes, they were incubated with the horseradish peroxidase-conjugated secondary antibody (Dianova, Hamburg, Germany). The blots were finally developed through enhanced chemiluminescence detection (Amersham Biosciences, IL, USA). A scanning laser densitometry (Biomed Instrument, Inc., CA, USA) was used to determine the intensities of the protein bands. Results were presented as arbitrary units relative to β-actin protein expression (Ragab et al. 2022).

### Quantitative real-time PCR

In each of SO, OVX/D-Gal, Donepezil, and *E. bonariensis* 100 mg/kg groups, total RNA was extracted from the left side of hippocampi samples (*n* = 5/group). The Direct-zol RNA Miniprep Plus (Cat# R2072, Zymo Research Corp., USA) was used according to the manufacturer’s instructions. Additionally, any possible contaminations of genomic DNA were removed via on-column DNA digestion, using an RNase free DNase kit. Then, the isolated total RNA was kept at – 80 °C. We determined the concentration of total RNA by Nano-drop 2000/c (Thermo Fisher Scientific, Wilmington, USA) and confirmed the presence of intact RNA using 2% agarose electrophoresis. Samples with clear 28 and 18S ribosomal RNA bands were selected for studying gene expression. The quantity and quality of RNA were assessed using Beckman dual spectrophotometer (USA). The isolated RNA was converted into cDNA using cDNA synthesis kit (SuperScript IV One-Step RT-PCR kit (Cat# 12594100, Thermo Fisher Scientific, Waltham, MA USA). Complementary DNA (cDNA) synthesis was performed according to the manufacturer's instructions. The expression patterns of α7-nAChR, Tau, PI3K, AKT, FOXO 3A, and GSK3B in addition to a housekeeping gene (GAPDH) were evaluated using the respective primers as described in Table 1. The real-time PCR reaction was performed in a thermal profile (48-well plate StepOne instrument; Applied Biosystem, USA) as follows: 10 min at 55 °C for reverse transcription, 2 min at 95 °C for RT inactivation and initial denaturation by 40 cycles of 10 s at 95 °C, 10 s at 55 °C and 30 s at 72 °C for the amplification step, then 5 min at 72 °C for final extension. The relative expression of the assessed genes was quantified versus GAPDH according to the 2^−∆∆CT^ method (Livak and Schmittgen 2001).

**Table 1:** Gene names and details of primers used for qRT-PCR analysis

Gene	Sequence 5ʹ to 3ʹ	Accession no.
*α7-nAChR*	F: CTT CAT GCA ACC AGG ATC AG R: TCT GTG CCC TTG ATA GCAC	S53987
*Tau*	F: ACGATTTCTGCTCCATGGTC R: AAGGTGACCTCCAAGTGTG	XM_039085764.1
*PI3K*	F: TTAAACGCGAAGGCAACGA R: CAGTCTCCTCCTGCTGTCGAT	XM_032898971.1
*AKT*	F: AATGACCGGGGAGTCCGAAT R: ATGTGCTTCATCCTGCCCAC	NM_001044712.1
*FOXO 3A*	F: GCCTCATCTCAAAGCTGGGT R: AGTTCTGCTCCACGGGAAAG	NM_001106395
*GSK3B*	F: AGCTGATCTTTGGAGCCACC R: TGGGGCTGTTCAGGTAGAGT	NM_032080
*GAPDH*	F: GTTACCAGGGCTGCCTTCTC R: GATGGTGATGGGTTTCCCGT	NM_017008

### Histological examination

The whole brain of two rats from each group was rapidly removed and fixed in freshly prepared 10 % neutral buffered formalin, processed routinely, and embedded in paraffin. Sections were cut in 5 μm-thickness and stained with hematoxylin and eosin (H and E) for blind histopathological examination and scoring under a light microscope (Mohamed et al. 2016).

### Statistical analysis

Results of the present study were analyzed using one-way ANOVA followed by Tukey’s multiple comparisons test and were expressed as mean ± SD. GraphPad Prism software (version 9) was used to perform the statistical analysis, with the level of significance being set at *p* < 0.05 for all statistical tests.

## Results

### Chemical composition of *E. bonariensis* extract

**Fig. 1:**
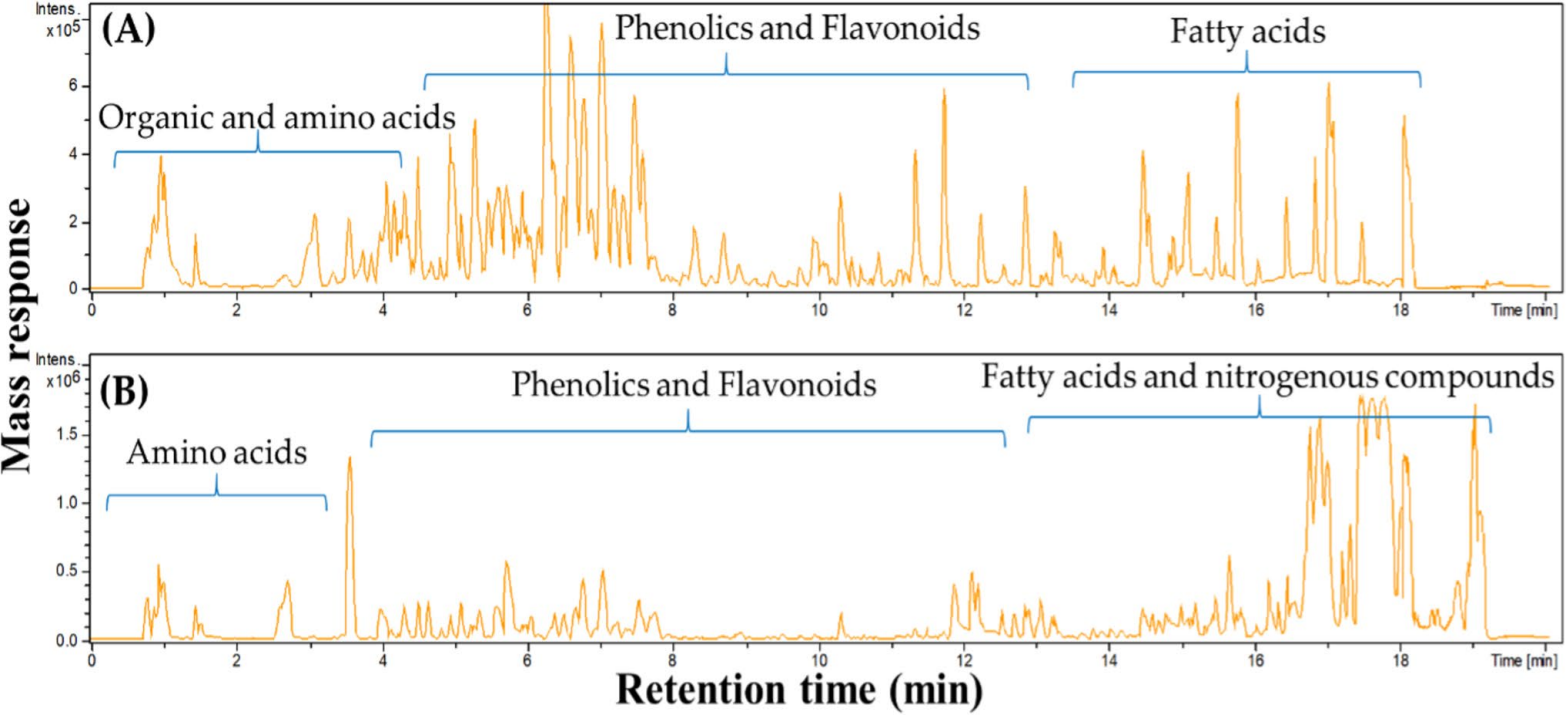
Representative UPLC–ESI–MS chromatogram of *E. bonariensis* alcohol extract carried out in **A** negative and **B** positive ionization modes

The chemical composition of *E. bonariensis* alcoholic extract was revealed utilizing UPLC–ESI-MS/MS (Fig. 1), operated in positive and negative ionization modes to provide a comprehensive overview of *E. bonariensis* metabolome. Assignment of metabolites was performed based on the comparison of mass fragmentation patterns to previously published data. A total of 42 chemicals were identified including 17 flavonoids, 6 phenolic acid derivatives, and 5 fatty acids, in addition to terpenoids and nitrogenous compounds as depicted in Table 2.

**Table 2:** Assigned metabolites in *E. bonariensis* alcohol extract via UPLC–ESI–MS analysis in negative and positive ion modes

RT [min]	Name	Class	Precursor	Formula	Error (ppm)	Fragments	Refs
0.967	Gluconic acid	Sugar acid	195.0511	C_6_H_11_O_7_^−^	0.3	177, 151, 129, 99, 87, 75	
1.002	Quinic acid	Organic acid	191.0557	C_7_H_11_O_6_^−^	− 2.1	127	
2.679	Phenylalanine	Amino acid	166.0864	C_9_H_12_NO_2_^+^	0.8	131, 120, 107, 103	
3.065	Dihydroxy-dimethyl-Oxobutyl-Alanine (Pantothenic acid)	Nitogenous compound	220.1183	C_9_H_18_NO_5_^+^	1.5	202, 184, 116, 90	(Pantami et al. 2020)
3.275	Xanthurenic acid	Quinoline	206.0446	C_10_H_8_NO_4_^+^	− 0.8	178, 160, 132	(An et al. 2018)
3.534	Tryptophan	Amino acid	203.0825	C_11_H_11_N_2_O_2_^−^	− 0.4	159, 142, 116, 74	
3.983	Caffeoyl-quinic acid	Phenolic	353.0874	C_16_H_17_O_9_^−^	− 1.1	191	
4.459	Apigenin-*C*-pentosyl-*C*-hexoside (Isoschaftoside)	Flavonoid	565.1553	C_26_H_29_O_14_^+^	0.2	547, 529, 505, 499, 475, 445, 427, 415, 409, 385, 356	(Keskes et al. 2018)
4.665	Luteolin-*C*-hexoside	Flavonoid	449.1074	C_21_H_21_O_11_^+^	− 0.9	431, 413, 383, 353,329, 299	(Afifi et al. 2023a)
4.82	*O*-Caffeoyl-quinic acid methyl ester (Methyl chlorogenate)	Phenolic	369.1184	C_17_H_21_O_9_^+^	1.1	177, 163	
5.044	Trihydroxy-flavone-*C*-hexoside (Isovitexin)	Flavonoid	433.1133	C_21_H_21_O_10_^+^	0.8	415, 313, 283	(Afifi et al. 2023b)
5.085	Apigenin-*O*-diglucuronide	Flavonoid	623.1248	C_27_H_27_O_17_^+^	0.8	447, 271	
5.581	Isorhamnetin-*O*-hexoside [2 M-H]	Flavonoid	955.2144	C_44_H_43_O_24_^−^	− 0.6	477	
5.65	Quercetin-*O*-hexoside (isoquercitrin)	Flavonoid	465.1025	C_21_H_21_O_12_^+^	− 0.5	303	
5.76	Kaempferol-*O*-glucuronide	Flavonoid	463.0872	C_21_H_19_O_12_^+^	0.2	287	
6.138	Dicaffeoylquinic acid (Cynarin)	Phenolic	515.1197	C_25_H_23_O_12_^−^	0.3	353, 191	(Fang et al. 2002)
6.183	Quercetin-*O*-malonylhexoside	Flavonoid	551.103	C_24_H_23_O_15_^+^	− 0.2	303, 231, 163	
6.281	Cimicifugic acid	Phenolic	449.1076	C_21_H_21_O_11_^+^	− 0.5	341, 303, 287, 273, 255, 193	(Li et al. 2003a)
6.656	Luteolin-*O-*hexoside	Flavonoid	447.0938	C_21_H_19_O_11_^−^	0.6	285	
7.18	Cynarin isomer	Phenolic	517.1337	C_25_H_25_O_12_^+^	− 0.6	163	(Fang et al. 2002)
7.214	Dicaffeoylquinic acid lactone	Phenolic	499.1231	C_25_H_23_O_11_^+^	− 0.7	337, 175	(El-Hawary et al. 2022)
7.463	Luteolin-*C*-hexoside isomer	Flavonoid	447.0934	C_21_H_19_O_11_^−^	0.8	357	
8.183	Trihydroxy-dimethoxyflavone-*O*-glucuronide (Tricin-*O*-glucuronide)	Flavonoid	507.1131	C_23_H_23_O_13_^+^	− 0.4	331, 315	
9.77	Trihydroxyflavone-*O*-dihexoside	Flavonoid	593.1528	C_27_H_29_O_15_^−^	2.7	431, 269	(Afifi et al. 2023a)
9.82	Unknown	Terpene	249.1481	C_15_H_21_O_3_^+^	− 1.6	231, 213, 203, 185, 177, 175	
10.287	Tetrahydroxyflavone (Luteolin)	Flavonoid	285.0415	C_15_H_9_O_6_^−^	3.6	241, 175	
10.673	Quercetin	Flavonoid	301.0347	C_15_H_9_O_7_^−^	2.17	285, 245, 179, 165, 151, 133, 121	(Hassan et al. 2023)
11.392	Trihydroxy-methoxy-flavone	Flavonoid	301.07	C_16_H_13_O_6_^+^	− 2.2	286, 283, 255, 241, 137	(Afifi et al. 2023b)
11.415	Costunolide	Terpene	233.153	C_15_H_21_O_2_^+^	− 7.3	215, 187, 177, 159	
12.021	Dihydroxy-Tetramethoxyflavone (Casticin)	Flavonoid	375.1061	C_19_H_19_O_8_^+^	− 3.5	360, 342, 317, 231, 215, 179	(Fu et al. 2020)
12.335	Dihydroxy-Dimethoxyflavone (Velutin)	Flavonoid	315.0856	C_17_H_15_O_6_^+^	− 2.2	300, 282, 257, 201, 187	(Gomes et al. 2022)
13.138	Sphingosine	Sphingolipid	300.2887	C_18_H_38_NO_2_^+^	− 3.3	282, 264, 252	(Saigusa et al. 2012)
14.463	Octadeca-tetraenoic acid	Fatty acid	277.2149	C_18_H_29_O_2_^+^	− 4.7	259, 135, 121	
15.034	Hydroxyoctadecadienoic acid	Fatty acid	295.2276	C_18_H_31_O_3_^−^	− 0.9	277, 195	
15.13	Unknown	Nitrogenous compound	305.1071	C_12_H_22_N_2_O_3_PS^+^	− 4	277, 249, 181, 169, 153	
15.223	Palmitoyl-glycero-phosphoethanolamine	Phospholipid	454.2925	C_21_H_45_NO_7_P^+^	− 0.6	436, 393, 313, 282, 216	(Neves et al. 2020)
15.457	Hydroxy-octadecatrienoic acid	Fatty acid	293.2123	C_18_H_29_O_3_^−^	0.2	275, 171	
15.752	Hydroxy-octadecatrienoic acid [2 M-H]	Fatty acid	587.4309	C_36_H_59_O_6_^−^	− 1.3	293	
16.319	Linoleamide	Nitrogenous compound	324.2888	C_18_H_34_NO^+^	− 2.7	307, 263, 245	
16.439	Icosatetraenoic acid (Arachidonic acid)	Fatty acid	305.2465	C_20_H_33_O_2_^+^	− 3.2	287, 259, 163, 149	
16.826	Docosenamide (Erucamide)	Nitrogenous compound	338.3406	C_22_H_44_NO^+^	− 3.3	321, 303	
17.236	Epoxylanostadienone (Cornusalterin L)	Terpene	439.3557	C_30_H_47_O_2_^+^	− 3	249, 203, 191	

#### Flavonoids

Flavonoids is considered the major metabolite class as represented by 17 entities mostly present as *O*- or *C*-glycosides. The *O*-glycosidic linkage, connecting the glycoside group to flavonoid aglycone, may easily be cleaved yielding product ions with 132, 146, 162, and 176 amu neutral mass losses indicating the presence of pentose, deoxyhexose, hexose, and hexuronyl moieties, respectively. In contrast, flavonoid-*C*-hexosides exhibited fragmentations at − 90, − 120, and – 150 amu due to sugar partial cleavages, while flavonoid-*C*-pentosides showed neutral loss at – 60 amu. Other common fragmentations of flavonoid aglycones involve retro Diels–Alder fission (Farag et al. 2022).

Peak 8 exhibited molecular ion at *m/z* 565.1553 [M+H]^+^ indicating a formula C_26_H_29_O_14_^+^, and product ions at *m/z* 505 [M+H-60]^+^, 475 [M+H-90]^+^, 445 [M+H-120]^+^, and 415 [M+H-150]^+^ suggesting *C*-pentosyl and *C*-hexosyl moieties. While, the other fragments at *m/z* 385 [M+H-180]^+^ and 356 [M+H-209]^+^, suggesting the aglycone linked to sugar residues, viz., apigenin (270) +115 and apigenin (270) + 86, respectively, indicated di-*C*-substituted flavone. Peak 8 was annotated as apigenin-*C*-pentosyl-*C*-hexoside (isoschaftoside) (Keskes et al. 2018) and reported in *Erigeron* genus for the first time. Likewise, peaks 9 and 11 revealed parent ions at *m/z* 449.1074 [M+H]^+^ C_21_H_21_O_11_^+^ and 433.1133 [M+H]^+^ C_21_H_21_O_10_^+^, respectively and fragment ions at [M+H-120]^+^ and [M+H-150]^+^ owing to *C*-hexosyl moieties. Peaks 9 and 11 were identified as luteolin-*C*-hexoside and trihydroxy-flavone-*C*-hexoside (isovitexin), respectively, and initially reported in *E. bonariensis*. Peaks 19 and 22 exhibited similar molecular ion at *m/z* 447.09 [M-H]^-^ C_21_H_19_O_11_^-^ albeit different fragmentations at *m/z* 285 [M-H-162]^-^ and 357 [M-H-90]^-^ assigning them as luteolin-*O*-hexoside and luteolin-*C*-hexoside isomer, respectively. Peak 24 (*m/z* 593.1528 [M-H]^-^ C_27_H_29_O_15_^-^) demonstrated consecutive losses of two *O*-hexosyl moieties at *m/z* 431 [M-H-162]^-^ and 269 [M-H-2×162]^-^ leading to its assignment as trihydroxyflavone-*O*-dihexoside. Such fragmentation pattern [M+H-162]^+^ was also observed in peak 14 (*m/z* 465.1025 [M+H]^+^ C_21_H_21_O_12_^+^) and annotated as quercetin-*O*-hexoside (isoquercitrin), previously reported in *E. bonariensis* (Zahoor et al. 2012). In addition, peak 13 (*m/z* 955.2144 [2M-H]^-^ C_44_H_43_O_24_^-^) was identified for the first time in *E. bonariensis* as dimer of isorhamnetin-*O*-hexoside. Furthermore, peak 17 (*m/z* 551.103 [M+H]^+^ C_24_H_23_O_15_^+^) exhibited daughter ion at *m/z* 303 [M+H-162-86]^+^ corresponding to the loss of *O*-hexosyl and malonyl moieties. Peak 17 is identified herein for the first time in *E. bonariensis* as quercetin-*O*-malonylhexoside. The fragmentation pattern of losing hexuronyl moiety (-176 amu) was detected in peaks 12, 15, and 23 and identified for the first time in *E. bonariensis* as apigenin-*O*-diglucuronide, kaempferol-*O*-glucuronide, and tricin-*O*-glucuronide, respectively.

Peak 30 (*m/z* 375.1061 [M+H]^+^ C_19_H_19_O_8_^+^) produced diagnostic fragment ions at *m/z* 360, 342 and 317 correlated to [M+H-methyl]^+^, [M+H-methyl-H_2_O]^+^, and [M+H-2methyl-CO]^+^, respectively. The identity of peak 30 was confirmed as dihydroxy-tetramethoxyflavone (casticin), in agreement with previously reported data (Fu et al. 2020). The fragmentation pattern of peak 31 was like casticin where peak 31 (*m/z* 315.0856 [M+H]^+^ C_17_H_15_O_6_^+^) revealed daughter ions at *m/z* 300 [M+H-methyl]^+^, 282 [M+H-methyl-H_2_O]^+^, and 257 [M+H-2methyl-CO]^+^, suggesting a polymethoxylated flavonoid, *viz.*, velutin (luteolin 7,3-dimethyl ether) (Gomes et al. 2022). Both casticin and velutin were reported herein for the first time in *Erigeron* genus. Other flavonoid aglycones were detected on negative ion mode in peaks 26 (*m/z* 285.0415 [M-H]^-^ C_15_H_9_O_6_^-^) and 27 (*m/z* 301.0347 [M-H]^-^ C_15_H_9_O_7_^-^) corresponding to tetrahydroxyflavone (luteolin) and quercetin, respectively. Quercetin was previously reported in *E. bonariensis* (Zahoor et al. 2012), while luteolin was detected in *Erigeron acris* (Nalewajko-Sieliwoniuk et al. 2019).

#### Phenolic acids

Six hydroxyl-cinnamic acid esters were detected for the first time in *E. bonariensis*, which are considered as potent antioxidants (Fraisse et al. 2011). In detail, five quinic acid esters were present in peaks 7, 10, 16, 20, and 21, belonging to *O*-caffeoyl-quinic acid derivatives characteristic to *Erigeron* (Zahoor et al. 2012). Peak 7 (*m/z* 353.0874 [M-H]^-^ C_16_H_17_O_9_^-^), characterized by loss of caffeoyl moiety at *m/z* 191 [M-H-162]^-^ corresponding to deprotonated quinic acid, was identified as *O*-caffeoyl-quinic acid. Another less polar peak 10 (*m/z* 369.1184 [M+H]^+^ C_17_H_21_O_9_^+^) revealed fragment ions due to neutral loss of quinic acid at *m/z* 177 [M+H-192]^+^ and protonated caffeoyl moiety at *m/z* 193 indicating *O*-caffeoyl-quinic acid methyl ester. Peaks 16 and 20 demonstrated fragment ions owing to the loss of two caffeoyl fragments (− 2×162) in sequence and were annotated as dicaffeoylquinic acid and its isomer, which were reported previously in *Erigeron acris* (Nalewajko-Sieliwoniuk et al. 2019). Likewise, peak 21 (*m/z* 499.1231 [M+H]^+^ C_25_H_23_O_11_^+^) showed same fragmentation pattern with two consecutive losses of caffeoyl fragments and was characterized as dicaffeoylquinic acid lactone. Finally, peak 18 (*m/z* 449.1076 [M+H]^+^ C_21_H_21_O_11_^+^) generated a base peak at *m/z* 255 [M+H-194]^+^ from neutral loss of ferulic acid in addition to a fragment ion at *m/z* 193 [M+H-194-H_2_O-CO_2_]^+^. Similarly, the formation of a daughter ion with less relative abundance at *m/z* 273 [M+H-176]^+^ was observed from neutral loss of feruloyl moiety, suggesting that this compound was cimicifugic acid (Li et al. 2003b).

#### Nitrogenous compounds

Several parent peaks revealed even molecular ions, suggesting the presence of nitrogenous atom. Peak 4 (*m/z* 220.1183 [M+H]^+^ C_9_H_18_NO_5_^+^) demonstrated successive loss of water at *m/z* 202 [M+H-18]^+^ and 184 [M+H-36]^+^. In addition, two daughter peaks at *m/z* 116 and 60 were detected, which were attributed to the cleavage of C*α*–CO and amidic bonds yielding acyl ion and protonated *β*-alanine, respectively. Peak 4 was annotated as pantothenic acid. Likewise, peak 32 (*m/z* 300.2887 [M+H]^+^ C_18_H_38_NO_2_^+^) suffered from successive loss of water at *m/z* 282 and 264 besides fragment at *m/z* 252 [M+H-18-30]^+^ corresponding to loss of one water and formaldehyde. Peak 32 was characterized as C18-sphingosine. Peak 5 (*m/z* 206.0446 [M+H]^+^ C_10_H_8_NO_4_^+^) showed fragments at *m/z* 178 [M+H-CO]^+^, 160 [M+H-CO-H_2_O]^+^, and 132 [M+H-CO-H_2_O-CO]^+^, and was identified as xanthurenic acid. Herein, pantothenic and xanthurenic acids and C18-sphingosine were reported for the first time in *Erigeron* genus. Peak 36 (*m/z* 454.2925 [M+H]^+^ C_21_H_45_NO_7_P^+^) revealed fragments at *m/z* 436 [M+H-H_2_O]^+^, 393 [M+H-H_2_O-C_2_H_5_N]^+^, 313 [M+H-141]^+^ due to the loss of phosphoethanolamine and *m/z* 216 [M+H-238]^+^ corresponding to loss of palmitoyl moiety. This peak was annotated as palmitoyl-glycero-phosphoethanolamine, initially reported in *E. bonariensis*. Peaks 39 (*m/z* 324.2888 [M+H]^+^ C_18_H_34_NO^+^) and 41 (*m/z* 338.3406 [M+H]^+^ C_22_H_44_NO^+^) were identified as linoleamide and erucamide for the first time in *Erigeron* genus. Both peaks showed similar fragmentation pattern owing to the loss of ammonia [M+H-17]^+^ and water [M+H-18]^+^.

### *E. bonariensis* extract ameliorated memory impairment in OVX/D-Gal-subjected rats in MWM test

**Fig. 2:**
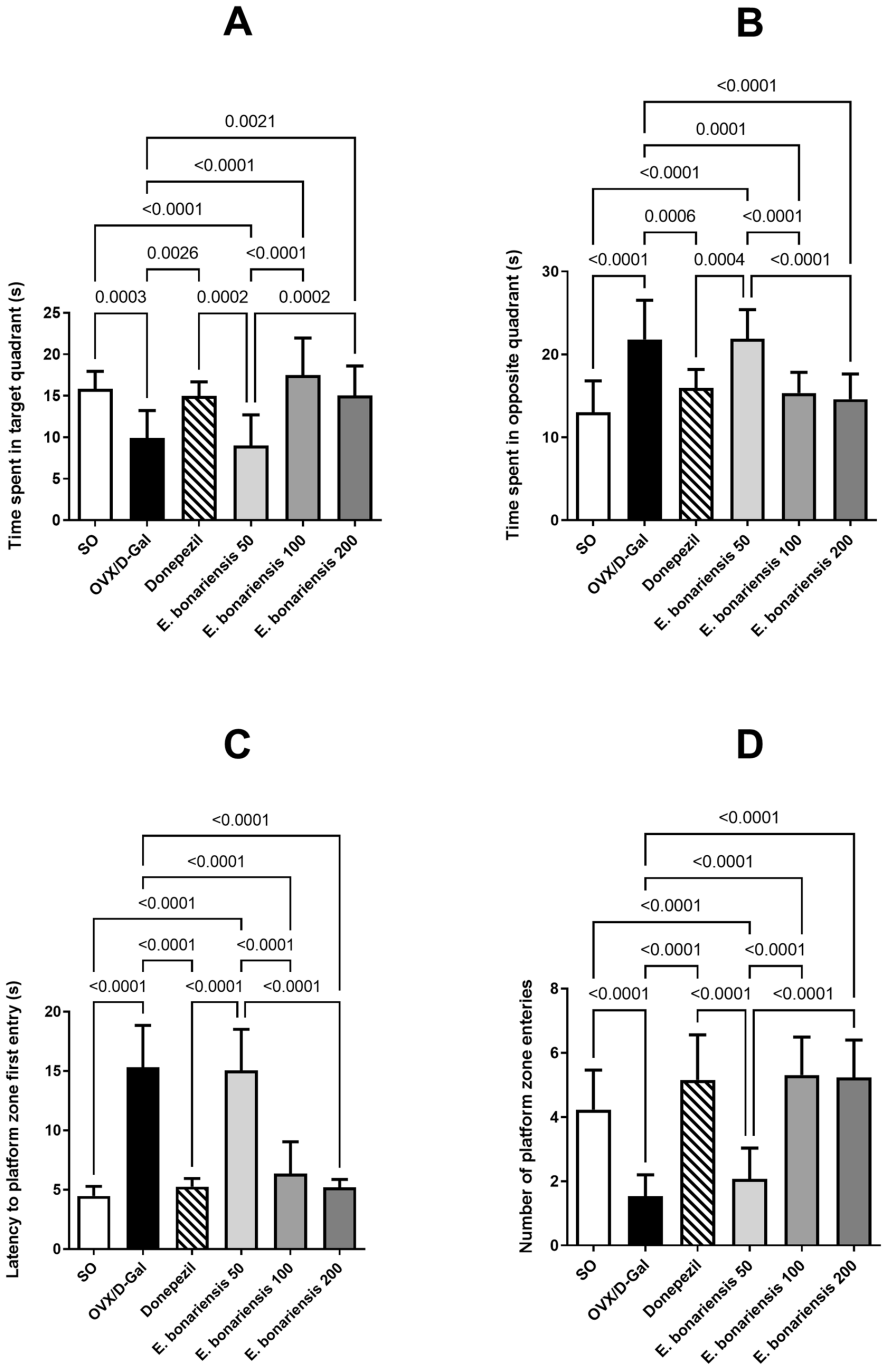
Effect of *E. bonariensis* on OVX/D-Gal-induced spatial memory deterioration in MWM test. **A** Time spent in the target quadrant, **B** TIME spent in the opposite quadrant, **C** latency to platform zone first entry, and **D** Number of platform zone entries. Rats underwent either SO or OVX, and after 5 days, they received D-Gal (150 mg/kg/day, i.p) for 42 days. OVX/D-Gal-subjected rats were orally treated with donepezil (5 mg/kg/day) or the alcoholic extract of *E. bonariensis* at three different doses (50, 100, and 200 mg/kg/day) for 42 days, given 1 h prior to D-Gal. Four days before the end of the experiment, all animals were subjected to MWM test where the training phase was conducted on days 39–41 and the probe trial was performed on day 42. Data were expressed as mean ± SD (*n* = 13), using one-way ANOVA followed by Tukey’s post hoc test (*P *< 0.05). OVX: ovariectomy, *D-Gal* D-galactose, *MWM* Morris water maze, *SO* sham operation

The effect of *E. bonariensis* extract on the spatial memory performance of OVX/D-Gal-subjected rats was evaluated at three different doses (50, 100, and 200 mg/kg/day) in the MWM. As described in Fig. 2, OVX/D-Gal rats spent less time in the target quadrant by 37% (*F*_(5, 72)_ = 1.730, *p* = 0.1386) contrary to more time in the opposite quadrant by 1.7-fold as compared to the SO group (*F*_(5, 72)_ = 0.9186, *p* = 0.4740). Additionally, rats took longer time to firstly enter the platform zone than the SO group rats by 3.4-fold (*F*_(5, 72)_ = 7.307, *p* < 0.0001) and exhibited reduced frequency of platform zone's entry by 64% (*F*_(5, 72)_ = 1.154, *p* = 0.3404). Administration of *E. bonariensis* extract in OVX/D-Gal rats at the doses of 100 and 200 mg/kg/day ameliorated their memory performance in a comparable manner. These doses succeeded in preventing OVX/D-Gal-induced memory impairment and restoring the values of all the previously mentioned behavioral variables to the normal range, producing equivalent effects to those of donepezil. However, the extract at a dose 50 mg/kg failed to exert any ameliorative effect on memory functions of OVX/D-Gal rats.

### *E. bonariensis* extract ameliorates histopathological alterations in OVX/D-Gal-subjected rats

**Fig. 3:**
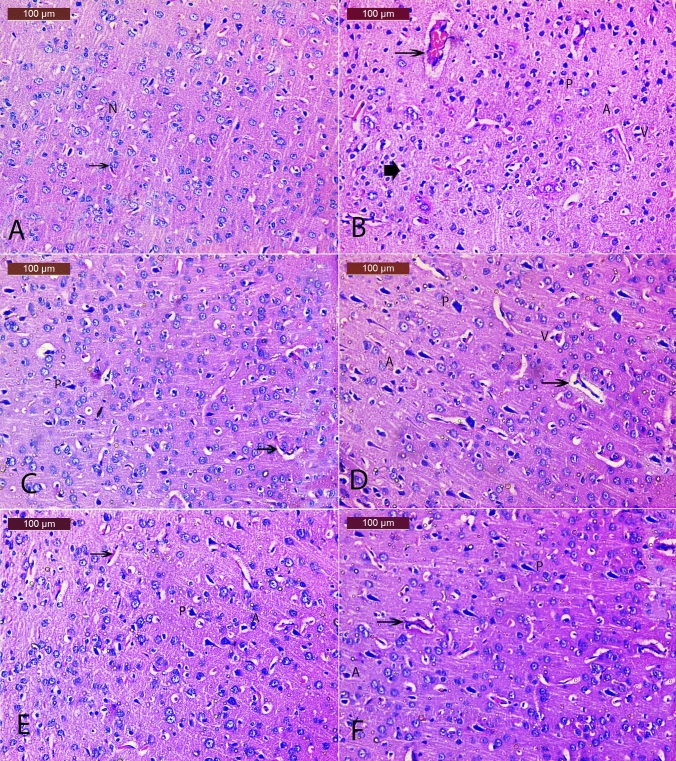
Effect of *E. bonariensis* on OVX/D-Gal-induced cerebral cortex histopathological changes. Representative H&E photomicrographs (cerebral cortex region) of all experimental groups (*n* = 2); magnification: Hand E × 200 **A** SO group showing normal structure of cerebral cortex and the neurons with their characteristic large vesicular nuclei (N); **B** OVX/D-Gal group showing numerous histopathological changes including a large number of damaged neurons, degenerated, necrotic (arrowhead), perineuronal vacuolation (V), pyknotic nuclei (P), apoptotic (A), and congestion of cerebral blood vessel (arrow); **C** Donepezil group showing nearly normal neuronal morphology with minimal pyknotic, apoptotic nuclei (P); **D** *E. bonariensis* 50 mg/kg group showing less histopathological changes except for pyknosis of some neurons (P), apoptotic nuclei, and perineuronal vacuolation (V); **E** *E. bonariensis* 100 mg/kg group showing almost normal histological structure. Some neurons had normally stained nuclei and other neurons showed minimal apoptotic cells (A) and pyknotic nuclei (P) with normal blood vessels (Bv); **F** *E. bonariensis* 200 mg/kg group showing almost normal neuronal cells of cortex with few histopathological changes such as minimal pyknotic (P) and apoptotic nuclei (A). Rats underwent either SO or OVX, and after 5 days, they received D-Gal (150 mg/kg/day, i.p) for 42 days. OVX/D-Gal-subjected rats were orally treated with donepezil (5 mg/kg/day) or the alcoholic extract of *E. bonariensis* at three different doses (50, 100, and 200 mg/kg/day) for 42 days, given 1 h prior to D-Gal. One day after behavioral testing (day 43), rats were decapitated, and the brains were separated for histopathological examination. *OVX* ovariectomy, *D-Gal* D-galactose, *SO* sham operation

In Table 3 and Fig. 3, cortical sections from the SO group showed normal histological structure as indicated by normally appearing neurons with prominent nucleoli (Fig. 3A). The OVX/D-Gal group showed severe histopathological alteration including a considerable number of injuries in neurons: degeneration, necrosis and perineuronal vacuolation, nuclei of the cells were shrunken, pyknotic, or apoptotic nuclei along with congestion of cerebral blood vessel (Fig. 3B). In contrast, cortical sections of Donepezil group exhibited nearly normal neuronal morphology with minimal pyknotic, apoptotic nuclei (Fig. 3C). Likewise, sections from *E. bonariensis* 50 mg/kg group showed moderate improvement as revealed by pyknosis of few neurons, less perineuronal vacuolation, few apoptotic nuclei and acidophilic cytoplasm, and mild congestion of cerebral blood vessels (Fig. 3D). However, cortical tissues of *E. bonariensis* 100 mg/kg group showed almost normal histological structure. Some neurons showed normally stained nuclei and other neurons showed minimal apoptotic cells and pyknotic nuclei with normal blood vessels (Fig. 3E). Sections of *E. bonariensis* 200 mg/kg group showed almost normal neuronal cells of the cortex. Still, a few histopathological changes such as minimal pyknotic and apoptotic nuclei with normal blood vessels were still seen (Fig. 3F).

**Table 3:** The severity of histological alterations in cortical tissues of histopathological alterations in OVX/D-Gal rats treated with *E. bonariensis* extract

Histopathological damage	SO	OVX/D-Gal	Donepezil	*E. bonariensis* (mg/kg)		
				50	100	200
Degeneration	−	+ + +	+	+ +	+	+ +
Perineuronal vacuolation	−	+ + +	−	+ +	+	+ +
Apoptosis	−	+ + +	−	+ +	+	+
Pyknotic nuclei	−	+ +	+	+ +	+	+ +

− Absent

+ Mild

+  + Moderate

+  +  + Severe

**Fig. 4:**
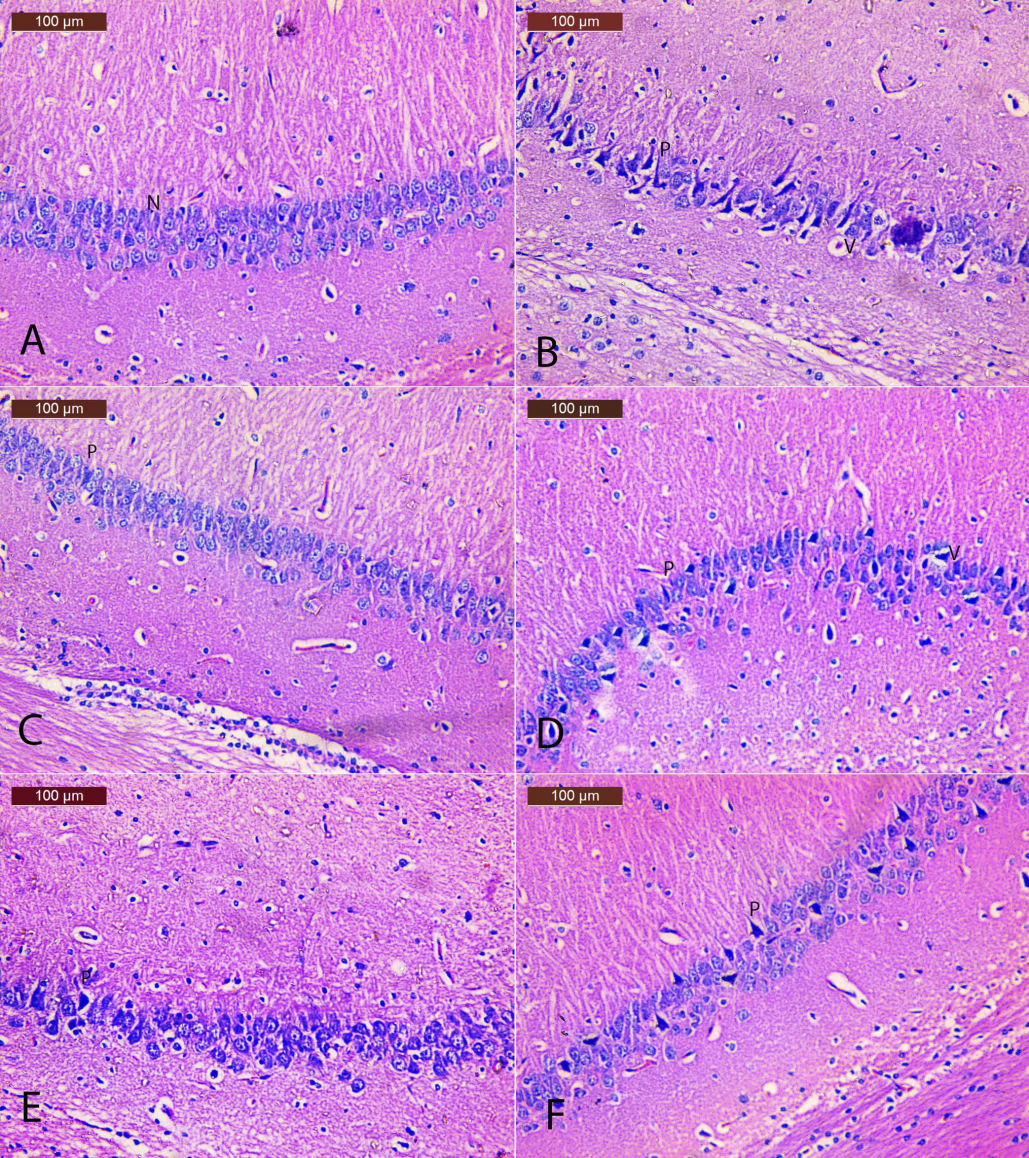
Effect of *E. bonariensis* on OVX/D-Gal-induced hippocampal histopathological changes. Representative H and E photomicrographs (hippocampal region) of all experimental groups (*n* = 2); magnification: H and E × 200 **A** SO group showing normal structure of hippocampus with normal structure of pyramidal cells (N); **B** OVX/D-Gal group showing vacuolated pyramidal cells (V) and pyknotic nuclei (P); **C** Donepezil group showing nearly normal architecture of the pyramidal cells of hippocampus with few pyknotic nuclei (P); **D** *E. bonariensis* 50 mg/kg group showing moderate vacuolated pyramidal cells (V) and pyknotic nuclei (P); **E** *E. bonariensis* 100 mg/kg group showing noticeable improvement of the hippocampus showed nearly normal appearance of most of the neurons, normal vesicular nucleoli. Some neurons showed minimal pyknotic nuclei (P); **F** *E. bonariensis* 200 mg/kg group showing nearly normal structure of pyramidal cells with mild pyknotic nuclei (P). Rats underwent either SO or OVX, and after 5 days, they received D-Gal (150 mg/kg/day, i.p) for 42 days. OVX/D-Gal-subjected rats were orally treated with donepezil (5 mg/kg/day) or the alcoholic extract of *E. bonariensis* at three different doses (50, 100, and 200 mg/kg/day) for 42 days, given 1 h prior to D-Gal. One day after behavioral testing (day 43), rats were decapitated, and the brains were separated for histopathological examination. *OVX* ovariectomy, *D-Gal* D-galactose, *SO* sham operation

In Table 4 and Fig. 4, hippocampal sections of the SO control group revealed normal architecture of the pyramidal cells (Fig. 4A). The OVX/D-Gal group showed numerous histopathological changes including many damaged neurons, degenerated pyramidal cells, and vacuolated neurons. Nuclei of the cells were shrunken, pyknotic, and hyperchromatic (Fig. 4B). The hippocampal sections of Donepezil group showed normal architecture of the pyramidal cells with few pyknotic nuclei (Fig. 4C). The sections of *E. bonariensis* 50 mg/kg group showed moderate tissue changes as mild vacuolated neurons with pyknotic nuclei (Fig. 4D), while those of *E. bonariensis* 100 mg/kg group showed noticeable improvement of the hippocampus as evidenced by a nearly normal appearance of most of the neurons and normal vesicular nucleoli. Some neurons showed pyknotic nuclei (Fig. 4E). The hippocampal sections of *E. bonariensis* 200 mg/kg group showed normal appearance of the hippocampal region and obvious improvement in most of the neurons and normal central vesicular nucleoli. Some neurons showed pyknotic nuclei (Fig. 4F).

**Table 4:** The severity of histological alterations in hippocampal tissues of OVX/D-Gal rats treated with *E. bonariensis* extract

Histopathological damage	SO	OVX/D-Gal	Donepezil	*E. bonariensis* (mg/kg)		
				50	100	200
Degeneration	−	+ +	+	+ +	+	+ +
Perineuronal vacuolation	−	+ +	−	+	+	+ +
Pyknotic nuclei	−	+ +	+	+ +	+	+ +

− Absent

+ Mild

+  + Moderate

+  +  + Severe

### *E. bonariensis* extract alleviated OVX/D-Gal-induced changes in AChE, Tau, and Aβ42 hippocampal levels

**Fig. 5:**
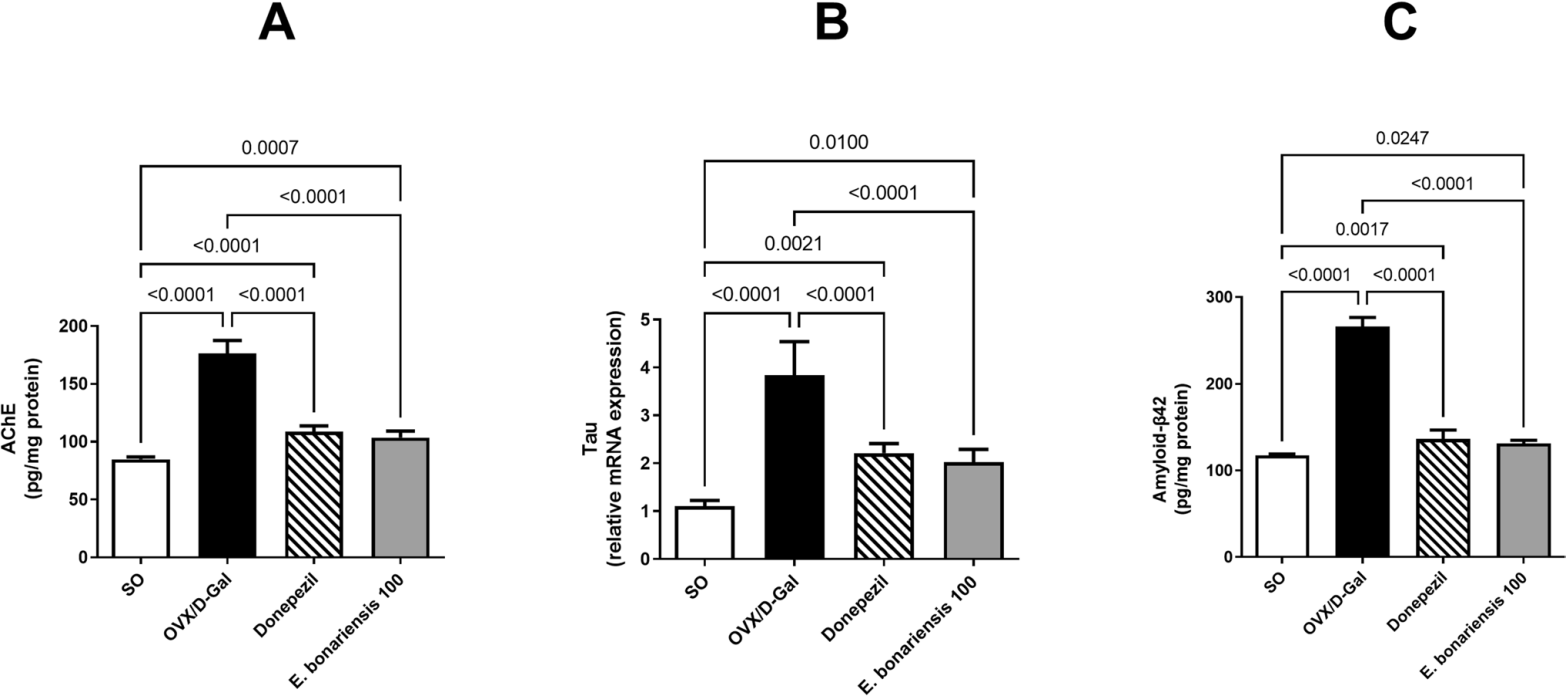
Effect of *E. bonariensis* on OVX/D-Gal-induced changes in hippocampal levels of **A** AChE, **B** Tau, and **C** Aβ42. Rats underwent either SO or OVX, and after 5 days, they received D-Gal (150 mg/kg/day, i.p) for 42 days. OVX/D-Gal-subjected rats were orally treated with donepezil (5 mg/kg/day) or the alcoholic extract of *E. bonariensis* (100 mg/kg/day) for 42 days, given 1 h prior to D-Gal. One day after behavioral testing (day 43), rats were decapitated, and the hippocampi were separated for biochemical analysis. Data were expressed as mean ± SD (n = 6 for AChE and Aβ42 concentrations, while *n* = 5 for Tau gene expression), using one-way ANOVA followed by Tukey’s post hoc test (*P* < 0.05). *OVX* ovariectomy, *D-Gal* D-galactose, *AChE* acetylcholinesterase, *Aβ42* amyloid-β42, *SO* sham operation

The intraperitoneal injection of D-Gal along with OVX caused a prominent elevation in the hippocampal levels of Tau (3.5-fold) and Aβ42 (2.3-fold), the hallmarks of AD, with a significant upsurge in the ACh hydrolyzing enzyme, AChE by 2.1-fold, in comparison with the SO group (for AChE: *F*_(3, 20)_ = 2.054, *p* = 0.1386; for Tau: *F*_(3, 16)_ = 3.105, *p* = 0.0562; for Aβ42: *F*_(3, 20)_ = 4.235, *p* = 0.0180). This was ameliorated by *E. bonariensis* extract (100 mg/kg/day) leading to a decrease in their levels by 47% (Tau), 51% (Aβ42), and 41% (AChE) as compared to the OVX/D-Gal group, and this effect was analogous to that produced by donepezil **(**Fig. 5**)**.

### *E. bonariensis* extract lessens OVX/D-Gal-induced changes in α7-nAChR, p-Jak2, and p-STAT3 hippocampal expression

**Fig. 6:**
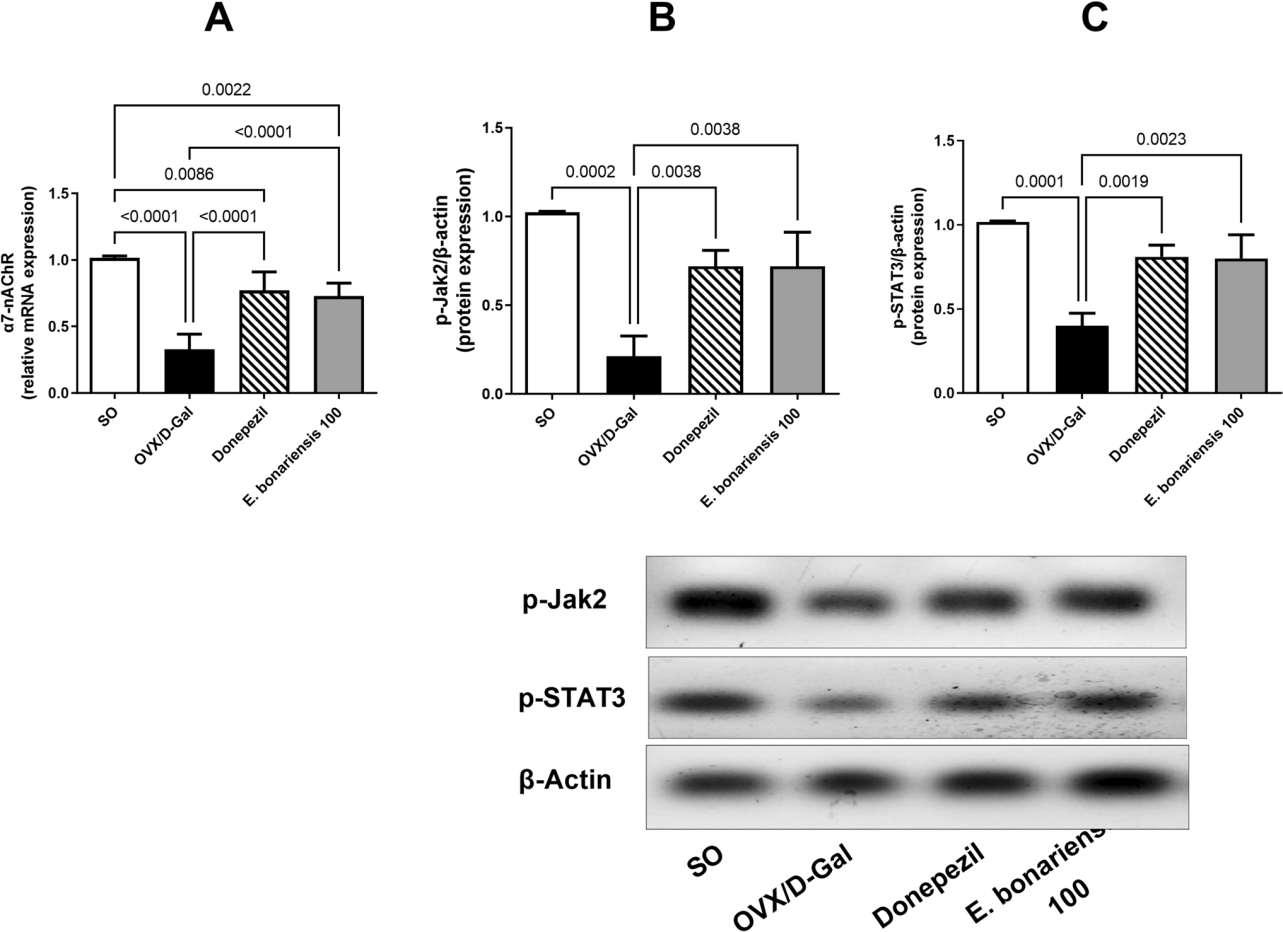
Effect of *E. bonariensis* on OVX/D-Gal-induced changes in hippocampal expression of **A** α7-nAChR, **B** p-Jak2, and **C** p-STAT3. Rats underwent either SO or OVX, and after 5 days they received D-Gal (150 mg/kg/day, i.p) for 42 days. OVX/D-Gal-subjected rats were orally treated with donepezil (5 mg/kg/day) or the alcoholic extract of *E. bonariensis* (100 mg/kg/day) for 42 days, given 1 h prior to D-Gal. One day after behavioral testing (day 43), rats were decapitated, and hippocampi were separated for biochemical analysis. Data were expressed as mean ± SD (*n* = 5 for α7-nAChR gene expression, while *n* = 3 for p-Jak2 and p-STAT3 protein expression), using one-way ANOVA followed by Tukey's post hoc test (*P* < 0.05). OVX: ovariectomy, D-Gal: D-galactose, Jak2: Janus kinase 2, SO; sham operation, STAT3: signal transducer and activator of transcription 3

In hippocampus, the key brain area responsible for learning and memory processing, α7-nAChR is extensively expressed. Its gene level in the hippocampi of the OVX/D-Gal group was significantly depressed by 68% as compared to the SO group (*F*_(3, 16)_ = 1.561, *p* = 0.2376). This was associated with a significant reduction in the hippocampal protein expression of p-Jak2 (79%) and p-STAT3 (61%), which are important effects of α7-nAChR (*F*_(3, 8)_ = 0.6681, *p* = 0.5950 for p-Jak2; *F*_(3, 8)_ = 1.254, *p* = 0.3532 for p-STAT3). The administration of *E. bonariensis* extract (100 mg/kg) in OVX/D-Gal-subjected rats restored the hippocampal α7-nAChR by 2.2-fold along with a rise in p-Jak2 and p-STAT3 hippocampal expression by 3.4- and 2-fold, respectively, producing a comparable effect with that of the donepezil group (Fig. 6).

### *E. bonariensis* extract modulated OVX/D-Gal-induced changes in PI3K, AKT, GSK-3β, and FOXO3a hippocampal expression

**Fig. 7:**
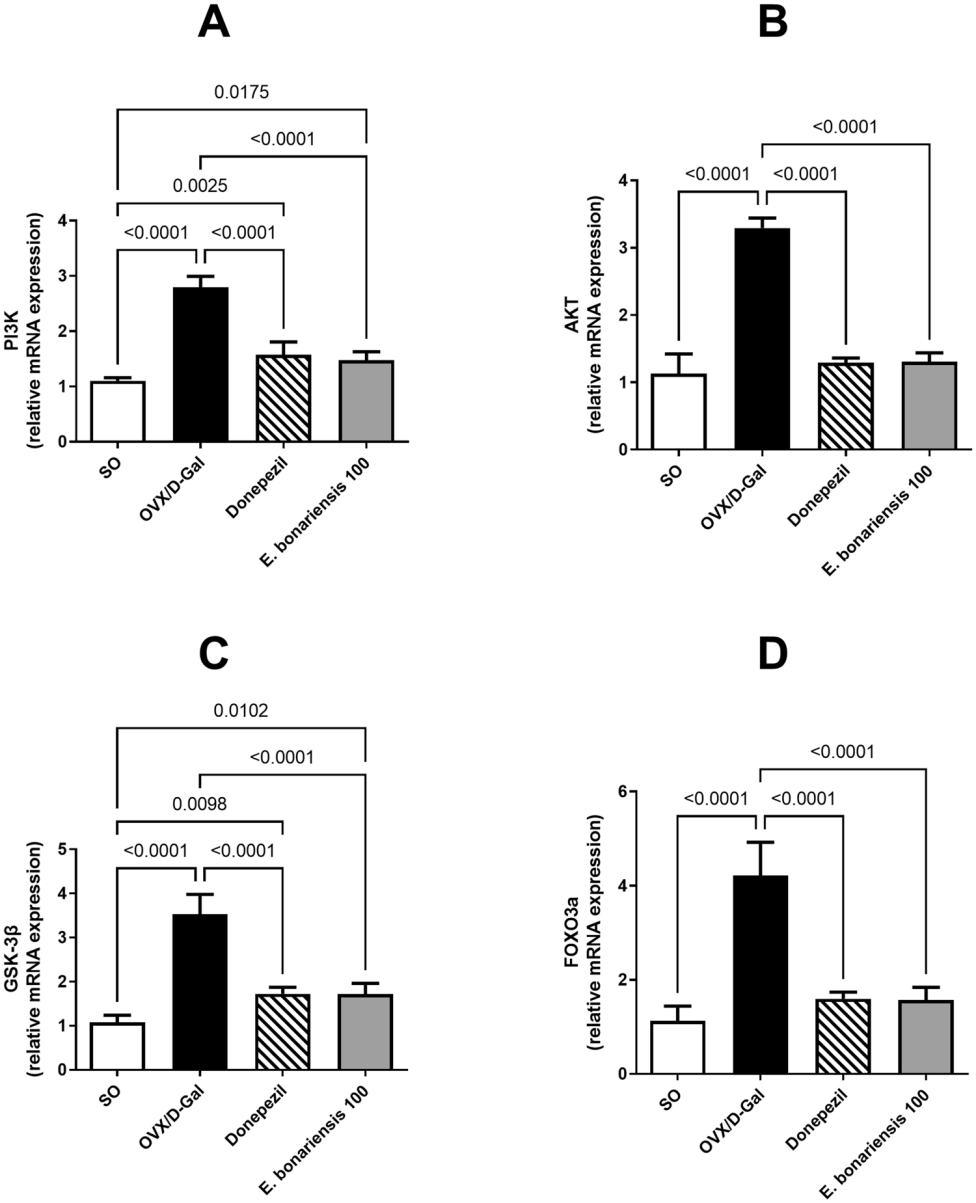
Effect of *E. bonariensis* on OVX/D-Gal-induced changes in hippocampal gene expression of **A** PI3K, **B** AKT,** C**GSK-3β, and** D** FOXO3a. Rats underwent either SO or OVX, and after 5 days they received D-Gal (150 mg/kg/day, i.p) for 42 days. OVX/D-Gal-subjected rats were orally treated with donepezil (5 mg/kg/day) or the alcoholic extract of *E. bonariensis* (100 mg/kg/day) for 42 days, given 1 h prior to D-Gal. One day after behavioral testing (day 43), rats were decapitated, and hippocampi were separated for biochemical analysis. Data were expressed as mean ± SD (*n* = 5), using one-way ANOVA followed by Tukey’s post hoc test (*P* < 0.05). *OVX* ovariectomy, *D-Gal* D-galactose, *PI3K* phosphoinositide-3 kinase, *Akt* protein kinase B, *GSK-3β* glycogen synthase kinase-3β, *FOXO3a* forkhead box O3, *SO* sham operation

The mRNA expression of PI3K, AKT, GSK-3β, and FOXO3a were significantly up-regulated in the hippocampi of OVX/D-Gal rats by 2.5-, 2.9-, 3.3-, and 3.7-fold, respectively, compared to SO rats (for PI3K: *F*_(3, 16)_ = 1.019, *p* = 0.4101; for AKT: *F*_(3, 16)_ = 1.924, *p* = 0.1664; for GSK-3β: *F*_(3, 16)_ = 1.560, *p* = 0.2380; for FOXO3a: *F*_(3, 16)_ = 2.180, *p* = 0.1302). Such effects were mitigated by treating the OVX/D-Gal rats with *E. bonariensis* at a dose of 100 mg/kg resulting in major reduction in their expression by 47% (PI3K), 60% (AKT), 51% (GSK-3β), and 63% (FOXO3a). Similarly, the reference drug donepezil significantly down-regulated the gene level of PI3K, AKT, GSK-3β, and FOXO3a producing analogous effects to that of *E. bonariensis* extract (100 mg/kg) (Fig. 7**).**

### *E. bonariensis* extract mitigated OVX/D-Gal-induced inflammation and apoptosis in rats

**Fig. 8:**
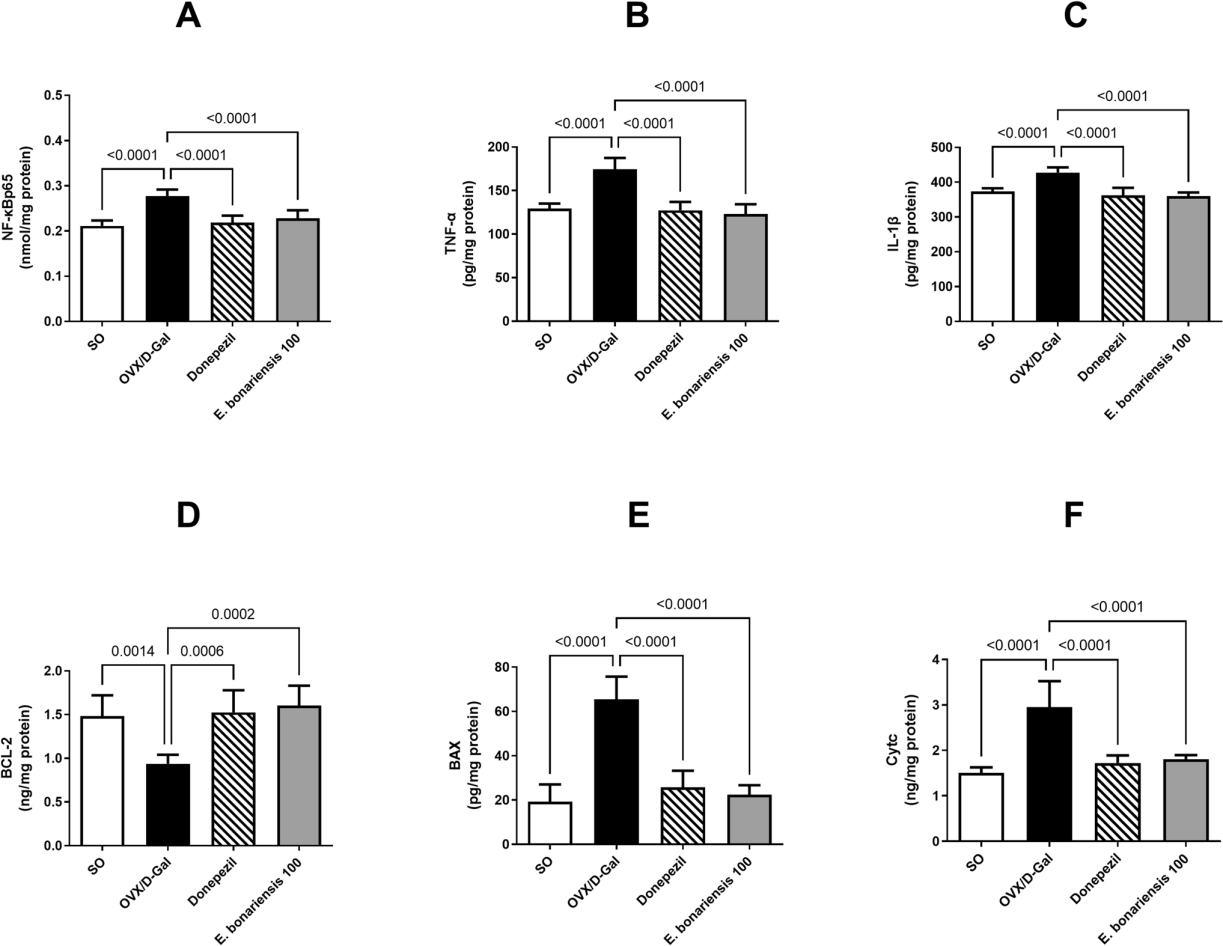
Effect of *E. bonariensis* on OVX/D-Gal-induced alterations in hippocampal contents** of (A)** BCL-2, **(B)** BAX, **(C)** Cytc, **(D)** NF-κBp65, **(E)** TNF-α, and **(F)** IL-1β. Rats underwent either SO or OVX, and after 5 days, they received D-Gal (150 mg/kg/day, i.p) for 42 days. OVX/D-Gal-subjected rats were orally treated with donepezil (5 mg/kg/day) or the alcoholic extract of *E. bonariensis* (100 mg/kg/day) for 42 days, given 1 h prior to D-Gal. One day after behavioral testing (day 43), rats were decapitated, and hippocampi were separated for biochemical analysis. Data were expressed as mean ± SD (n = 6), using one-way ANOVA followed by Tukey’s post hoc test (*P* < 0.05). *OVX* ovariectomy, *D-Gal* D-galactose, *BCL-2* B-cell lymphoma 2, *BAX* BCL-2 associated X, *Cytc* cytochrome C, *NF-κBp65* nuclear factor- κBp65, *TNF-α* tumor necrosis factor-α, *IL-1β* interleukin-1β, *SO* sham operation

Ovarian excision combined with D-Gal administration led to a profound state of apoptosis as well as aggravated neuroinflammatory response. This was evidenced by a prominent elevation in the hippocampal content of the pro-inflammatory markers and cytokines, namely, NF-κBp65 (1.3-fold), TNF-α (1.4-fold), and IL-1β (1.1-fold) in the OVX/D-Gal group as compared to the SO group (for NF-κBp65: *F*_(3, 20)_ = 0.1082, *p* = 0.9543; for TNF-α: *F*_(3, 20)_ = 1.085, *p* = 0.3782; for IL-1β: *F*_(3, 20)_ = 0.6984, *p* = 0.5640). Likewise, the OVX/D-Gal group demonstrated a remarkable depression in the hippocampal content of BCL-2, the anti-apoptotic marker by 37%, along with significant increase in levels of the pro-apoptotic markers, BAX and Cytc by about 3.4- and 2-fold, respectively, in comparison with their control counterparts (for BCL-2: *F*_(3, 20)_ = 1.074, *p* = 0.3824; for BAX: *F*_(3, 20)_ = 1.886, *p* = 0.1645; for Cytc: *F*_(3, 20)_ = 8.497, *p* = 0.0008). Such effects were mitigated upon *E. bonariensis* extract administration at the dose of 100 mg/kg, in an equivalent manner to that of donepezil, prominently reducing the NF-κBp65 (18%), TNF-α (30%), IL-1β (16%), Cytc (39%), and BAX (66%), while increasing the BCL-2 levels (1.7-fold), as compared to the OVX/D-Gal group (Fig. 8).

## Discussion

The current study is the first to illustrate the effectiveness of *E. bonariensis* extract in mitigating cognitive decline and AD-like pathological alterations in OVX/D-Gal rats. This finding is supported by (i) an improvement in spatial memory of rats; (ii) attenuation of OVX/D-Gal-induced histopathological alterations; (iii) reduction of Aβ1-42 and p-Tau, the disease hallmarks; (iv) stimulating the cholinergic activity; (v) increase in the expression of α7-nAChRs; (vi) modulation of Jak2/STAT3/NF-ĸB p65 and PI3K/AKT signaling cascades; (vii) down-regulating the expression of GSK-3β and FOXO3a; and (viii) the anti-inflammatory and anti-apoptotic activities.

In the present study, OVX/D-Gal group rats exhibited spatial learning memory impairments, as evidenced by the results of the MWM test, which is in line with former findings (Kamel et al. 2018; Ibrahim et al. 2019, 2023). However, treatment with *E. bonariensis* extract (100 or 200 mg/kg) markedly improved the memory performance of OVX/D-Gal group rats, producing effects equivalent to those of donepezil. This suggests the memory-protective potential of the extract in AD. These results were consistent with the histopathological analysis, which demonstrated that *E. bonariensis* extract successfully preserved the cerebral cortex and hippocampus tissues of OVX/D-Gal group rats.

The Aβ42 aggregation and formation of neurofibrillary tangles containing hyperphosphorylated Tau protein are major culprits in the pathogenesis of AD (Zhang et al. 2021; El-Hawary et al. 2021). These neurotoxic pathological hallmarks are responsible for cognitive decline, significant inflammatory response, synaptic dysfunction, and neuronal death (Kolarova et al. 2012; Sadigh-Eteghad et al. 2015). Herein, OVX/D-Gal induced a marked elevation in the hippocampal level of Aβ42 and expression of p-Tau. However, treatment with *E. bonariensis* extract (100 mg/kg) ameliorated the aforementioned effects, supporting its neuroprotective role.

Our results also show that OVX/D-Gal group rats demonstrated an upsurge of hippocampal AChE content with subsequent cognitive and memory impairments, as reported previously (Abdelkader et al. 2020). Treatment with *E. bonariensis* extract (100 mg/kg) succeeded in boosting the cholinergic activity through the profound decrease of hippocampal AChE content in OVX/D-Gal group rats. In agreement, a previous study reported the cholinomimetic activities of ethanolic and chloroform extracts of *C. bonariensis* (Yaseen et al. 2014). Furthermore, various plant extracts have begun to gain attention as potential inhibitors of AChE that could be used as a therapeutic option for AD (Taqui et al. 2022). Herein, the detected polyunsaturated fatty acids, i.e., arachidonic acid, conjugated linolenic, and linoleic acids in *E. bonariensis* extract, can impact the cholinergic system. It was reported that the presence of polyunsaturated fatty acids is necessary for effective cholinergic transmission (Lesa et al. 2003). In addition, flavonoids and other polyphenol compounds can reverse motor and cognitive deficits in aging (Ramezani et al. 2023). It was reported that kaempferol-*O*-glucuronide, luteolin, quercetin, and isoquercitrin, detected in *E. bonariensis* extract using LC-MS as peak no. 15, 26, 27 and 14, respectively (Table 2), have the ability to inhibit the activity of AChE leading to the improvement of signal transmission in cholinergic neurons’ synapses (Balkis et al. 2015). Moreover, those particular flavonoids revealed potent anti-AChE activity than other flavonoids lacking free OH group at position 4 of ring B (Khan et al. 2018). Noteworthy, xanthurenic acid detected in *E. bonariensis* extract peak no. 5 was reported to impede the transport of glutamate into synaptic vesicles, thus reducing glutamatergic transmission and, ultimately, lowering glutamate release at the synaptic level (Fazio et al. 2017). However, this effect was outweighed by major polyunsaturated fatty acids, flavonoids, and other polyphenol compounds detected in *E. bonariensis* extract.

Accumulating evidence suggests the role of α7-nAChRs in the pathogenesis of cognitive dysfunction in AD (Ma et al. 2014). Aβ42 binds to α7-nAChRs with high affinity, reducing the expression of the receptor and impairing learning and memory (Karthick et al. 2019; Tofighi et al. 2021). Furthermore, α7-nAChRs present in immune cells are the primary receptors in the “anti-inflammatory cholinergic pathway” (Hoskin et al. 2019). Activation of α7-nAChRs has been reported to inhibit lipopolysaccharide (LPS)-induced cognitive dysfunction and neuroinflammation in the hippocampus of mice (Alzarea and Rahman 2019). The derangement of the Jak/STAT pathway has been implicated in neuroinflammation and neuronal survival (Campbell 2005). The α7-nAChR activation inhibits NF-κB p65 activity by stimulating the Jak2/STAT3 signaling cascade, ultimately suppressing the production of pro-inflammatory cytokines (Marrero and Bencherif 2009; Egea et al. 2015). In the present study, treatment with *E. bonariensis* extract (100 mg/kg) up-regulated α7-nAChR mRNA expression, Jak2, STAT3 expression in hippocampus of OVX/D-Gal group rats, along with consequent suppression of NF-ĸB p65 level. These findings suggest the role of α7-nAChR activation in the neuroprotective and cognitive-enhancing effects of *E. bonariensis* extract via modulating the Jak2/STAT3, a signaling pathway that negatively regulates NF-κB p65. Of note, certain flavonoids or phenolic acids, such as luteolin (Parker-Athill et al. 2009), quercetin (Wu et al. 2019), cirsimaritin (Lee et al. 2016), and caffeoyl-quinic acid (Kour et al. 2022), are capable of modulating Jak2/STAT3 signaling.

The other arm upon which *E. bonariensis* alcoholic extract acted to attenuate OVX/D-Gal-induced neuroinflammation and cognitive impairment is the PI3K/AKT signaling pathway. The PI3K/AKT signaling pathway has been proven to play an important role in many physiological processes of the CNS, such as cell survival, neurogenesis, synaptic plasticity, and apoptosis (Long et al. 2021). Agonists of α7 nAChR are reported to stimulate phosphorylation of AKT via activation of Jak2 and PI3K (de Jonge and Ulloa 2007). Once PI3K/AKT pathway is activated, it ameliorates neuroinflammation *via* inhibiting the downstream effectors GSK-3β and FOXO3a (Matsuo et al. 2018). GSK-3β induces the production of pro-inflammatory cytokines through activation of NF-ĸB and promotes Tau phosphorylation and neuronal apoptosis (Wang et al. 2010; Martin et al. 2011). Moreover, GSK-3 negatively influences the learning and memory processes by delaying the induction of long-term potentiation (Peineau et al. 2007). FOXO3a is a key regulator of apoptosis that promotes Aβ-induced neurotoxicity (Qin et al. 2008). Activated AKT phosphorylates and inactivates GSK-3β and FOXO3a (Maiese 2016; Yang et al. 2020). The findings of the current study showed that treatment with *E. bonariensis* extract (100 mg/kg) augmented PI3K and AKT expression in hippocampus of OVX/D-Gal rats along with down-regulating the expression of GSK-3β and FOXO3a. These results suggest that PI3K/AKT signaling pathway is involved in neuroprotection by *E. bonariensis* extract in OVX/D-Gal-induced neurotoxicity via α7-nAChR stimulation. Certain flavonoids or phenolic acids, such as cimicifugic acid (Wang et al. 2017), caffeoyl-quinic acid (Yang et al. 2022), casticin (Fan et al. 2018), cirsimaritin (Kim et al. 2015), tricin (Liu et al. 2022), quercetin (Tu et al. 2021), and luteolin (Park and Song 2013), are capable of modulating PI3K/AKT signaling, of which several were detected as major components in *E. bonariensis* extract using LC–MS with peak no. 18, 7, 30, 23, 27, and 14, respectively (Table 2).

The association of neuroinflammation with AD is well known and is provoked via Aβ aggregation, resulting in microglial activation in hippocampal tissues (Glass et al. 2010). The crosstalk between Jak2/STAT3/NF-ĸB p65 and PI3K/AKT can provide the credential for the *E. bonariensis* extract-induced reduction of the inflammatory cascade observed herein, indicated by the reduced levels of the pro-inflammatory cytokines TNF-α and IL-1β. In agreement, it has been reported that *E. bonariensis* extract attenuated LPS-induced depressive-like behavior in mice through impeding neuroinflammation (Barua et al. 2019). This promising anti-inflammatory activity could be offered by the fatty acids as represented by arachidonic, linoleic, and linolenic acids with a reported role to suppress LPS-induced expression of COX-2 in macrophages by inhibiting NF-kB expression (Tortosa-Caparrós et al. 2017). Further, numerous mechanisms were hypothesized regarding the potential of flavonoids for decreasing Aβ accumulation (Hole and Williams 2021). Flavonoids can reduce Aβ production (Uddin et al. 2020), suppress GSK-3β-mediated Tau phosphorylation (Pierzynowska et al. 2019), and directly inhibit aggregation (Hole and Williams 2021).

Furthermore, enhanced neuronal apoptosis is also highly correlated with AD (Choi et al. 2023). The Bcl-2 family proteins have been postulated as the key regulators of mitochondria-mediated apoptosis and are implicated in neuronal apoptosis (Li et al. 2016). Bcl-2 family members are classified into those that protect cells from apoptosis (e.g., Bcl-2), and those that induce apoptosis (e.g., Bax) (Sayed et al. 2016). Moreover, apoptotic stimuli induce the release of Cyt C into the cytosol from the mitochondria (Zhang et al. 2018). Piling evidence exists toward numerous apoptotic insults of neuronal cells involved in the downregulation of Bcl-2 and up-regulation of Bax (Mattioli et al. 2005). The α7 nAChR/Jak2/STAT3 signaling is reported to induce the production of the anti-apoptotic protein Bcl2 in Aβ-induced apoptosis of PC12 cells (Marrero and Bencherif 2009). Our findings showed that *E. bonariensis* extract (100 mg/kg) increased Bcl-2, while suppressing Bax and Cyt C levels in the hippocampus of OVX/D-Gal rats. These findings reveal that *E. bonariensis* extract could inhibit neuronal apoptosis in via α7-nAChR activation.

## Conclusion

The current study demonstrates the neuroprotective and memory-enhancing capacity of *E. bonariensis* extract in the OVX/D-Gal rat model of AD through increasing α7-nAChRs expression and modulating Jak2/STAT3/NF-ĸB p65 and PI3K/AKT signaling cascades. Treatment with *E. bonariensis* extract also alleviated Aβ aggregation, tau hyperphosphorylation, neuroinflammation, and apoptosis caused by OVX and D-Gal administration. Thus, *E. bonariensis* extract may be a promising candidate for the management of AD. Identification of the most active fraction or isolating active chemicals in that complex mixture of *E. bonariensis* extract should now follow, alongside standardization to promote its use as future nutraceutical for neurodegenerative diseases.

## Data Availability

The datasets generated during and/or analyzed during the current study are available from the corresponding author on reasonable request.

## Abbreviations

AD: Alzheimer disease
UPLC–ESI-MS: Ultra-performance liquid chromatography–electrospray ionization-tandem mass spectrometry
D-Gal: D-galactose
E. bonariensis: *Erigeron bonariensis*
OVX: Ovariectomy
SO: Sham operation
MWM: Morris water maze test
α7-nAChRs: Alpha 7 Nicotinic acetylcholine receptors
NF-ĸB: Nuclear factor kappa-light-chain-enhancer of activated B cells
I.P.: Intraperitoneal injection
AChE: Acetylcholinesterase
GAPDH: Glyceraldehyde 3-phosphate
Aβ: Amyloid-β
